# LDL-Cholesterol Increases the Transcytosis of Molecules through Endothelial Monolayers

**DOI:** 10.1371/journal.pone.0163988

**Published:** 2016-10-03

**Authors:** Ana Magalhaes, Inês Matias, Inês Palmela, Maria Alexandra Brito, Sérgio Dias

**Affiliations:** 1 Instituto de Medicina Molecular, Faculdade de Medicina da Universidade de Lisboa, Lisboa, Portugal; 2 Research Institute for Medicines, Faculdade de Farmácia, Universidade de Lisboa, Lisboa, Portugal; 3 Department of Biochemistry and Human Biology, Faculdade de Farmácia, Universidade de Lisboa, Lisboa, Portugal; Hungarian Academy of Sciences, HUNGARY

## Abstract

Cholesterol has been identified as a causative factor in numerous pathologies including atherosclerosis and cancer. One of the frequent effects of elevated cholesterol levels in humans is the compromise of endothelial function due to activation of pro-inflammatory signalling pathways. While the mechanisms involved in endothelial activation by cholesterol during an inflammatory response are well established, less is known about the mechanisms by which cholesterol may affect endothelial barrier function, which were the subject of the present study. Here we show that low density lipoprotein (LDL) increases the permeability of endothelial monolayers to high molecular weight dextrans in an LDL receptor and cholesterol-dependent manner. The increased permeability seen upon LDL treatment was not caused by disruption of cell-to-cell junctions as determined by a normal localization of VE-Cadherin and ZO-1 proteins, and no major alterations in transendothelial electrical resistance or permeability to fluorescein. We show instead that LDL increases the level of high molecular weight transcytosis and that this occurs in an LDL receptor, cholesterol and caveolae-dependent way. Our findings contribute to our understanding of the systemic pathological effects of elevated cholesterol and the transport of cargo through endothelial monolayers.

## Introduction

The endothelium forms a barrier to the free passage of molecules and cells from the blood to tissues and vice-versa [1]. Therefore, crossing the endothelium is a tightly controlled process that may have pathological consequences if compromised. In fact endothelial dysfunction is an early finding in the course of atherosclerosis and cancer, and has increasingly been recognized in neurodegenerative diseases, such as Alzheimer’s disease [2,3,4]. Conversely, reduced or limited endothelial barrier permeability, such as that present in the blood-brain barrier, can reduce drug delivery and thus limit therapeutic interventions [5]. Endothelial barrier function is achieved by the presence of specialized cell-to-cell junctional complexes, including adherens and tight junctions, which tightly regulate the passage of molecules and cells across endothelia by the paracellular route. Endothelial cells also present a vesicular system of apical to basal transport that delivers cargo to tissues by the transcellular route or transcytosis [1].

Cholesterol is a component of cellular membranes where it exerts structural functions and acts as a platform for the interaction of signalling molecules in the so-called lipid rafts [6]. Hypercholesterolemia, the presence of high cholesterol levels in the blood, is a well characterized risk factor for atherosclerosis and has also more recently been shown to be involved in other diseases such as cancer and neurodegenerative diseases [7,8,9,10,11,12,13]. Cholesterol is carried in the blood by lipoproteins including low density lipoprotein (LDL) and the pathological effects of hypercholesterolemia are mainly linked to increased levels of LDL in circulation [8,14,15]. LDL delivers cholesterol to cells and undergoes post-translational modifications while in circulation such as oxidation. There are several types of membrane LDL receptors that bind native and modified LDL (oxidized or acetylated). Among them is the LDL receptor (LDLR), which binds native LDL (nLDL) [16].

Alterations in endothelial permeability in atherosclerosis prone areas of large arteries have been attributed mainly to the action of oxidized LDL (oxLDL), shown to accumulate in atherogenic plaques [3,17]. *In vitro*, early studies have shown that oxLDL increases endothelial permeability by inducing cell damage and disrupting cell-to-cell junctions [18,19]. Here using nLDL we detect no alterations on permeability at the level of the paracellular route but instead we see increased transport of high molecular weight molecules through endothelial cells. In addition we show that this is LDLR, cholesterol and caveolae-dependent.

## Material and Methods

### Antibodies and Reagents

All reagents were obtained from Sigma unless stated otherwise. Human plasma low density lipoprotein (LDL) was obtained from Calbiochem (Gibbstown, NJ, USA). The antibodies used were the following: anti-LDLR from R&D systems, anti-VE-Cadherin from Santa Cruz, anti-ZO-1 from Invitrogen, anti-Caveolin-1 from Santa Cruz and anti-Clathrin Heavy Chain from BD Biosciences. Alexa Fluor 488 secondary antibody was obtained from Invitrogen and VECTASHIELD mounting media from Vector Laboratories. HRP-conjugated secondary antibodies were purchased from Vector Laboratories and on-target plus siRNAs from Dharmacon.

### Cell culture

Human umbilical vein endothelial cells (HUVECs) (Lonza) were cultured in complete EBM-2 medium (Lonza) supplemented with 10% fetal bovine serum (FBS) on 0.2% gelatin-coated plates under a 37°C and 5% CO_2_ atmosphere. Passages three to five were used in all experiments.

### Transendothelial permeability assay to dextrans

Permeability to high molecular weight dextrans was performed as described earlier [20]: 5x 10^4^ HUVECs were plated on top of 0.2% gelatin coated transwells (6.5 mm inserts and 0.4 μm pores from Corning) (Day1). Cells were grown on transwells for 7 days in order to form a confluent and mature monolayer. Media was replaced the day after plating (Day 2) and 4 days after plating (Day4) with 600 μl of complete EBM-2 medium without serum (SFM) at the bottom and 100 μl of complete EBM-2 medium supplemented with 10% FBS at the top. Six days after plating both bottom and top media was replaced by SFM (Day 6) and on the following day cells were treated with the indicated conditions (Day 7). At day 8 media was replaced once more by SFM on both chambers and 1mg/ml of 70 kDa FITC- dextrans was added to the top chamber either straight after the washing or two hours after the washing. When indicated, inhibitors were also added before the dextrans. At indicated time points 50 μl of media were collected from the bottom chamber and analyzed for fluorescence emission with excitation at 488 nm and emission at 520 nm using a microplate reader (Tecan: Infinite M200). When samples were taken at several time points, 50 μl of fresh media were added to the bottom chamber to replace the volume that had been taken. For the transcytosis studies, dextrans were added to the top chamber for 15 minutes after which both top and bottom chamber were washed. 50 μl of media were collected from the bottom chamber and analyzed for fluorescence emission.

### TEER

For transendothelial electrical resistance (TEER) measurements 1.5 x 10^5^ cells were cultured in 12 mm diameter transwell inserts (Corning) and treated as described above. At the end of the assay, measurements were performed using an EndOhm™ chamber coupled to an EVOMX resistance meter (World Precision Instruments, Inc., USA). Results are shown in relation to average control readings, after deducting the empty insert values.

### Permeability to fluorescein

Permeability to sodium fluorescein (NaF, molecular weight: 376 Da) was determined as described previously [21]. Briefly, 1.5 x 10^5^ cells were cultured in 12 mm diameter transwell inserts and treated as described above. At the end of the assay the inserts were transferred to 12-well plates containing Ringer–Hepes solution (118 mM NaCl, 4.8 mM KCl, 2.5 mM CaCl_2_, 1.2 mM MgSO_4_, 5.5 mM d-glucose, 20 mM Hepes, pH 7.4). The NaF solution (10 mg/ml NaF in Ringer–Hepes solution) was then added to the top chambers. The inserts were transferred to new wells at 20, 40, and 60 min. Bottom chamber solutions were collected and analyzed for fluorescence emission with excitation at 440 nm and emission and 525 nm using a plate reader. Flux across cell-free inserts was also measured. The endothelial permeability coefficient *Pe* was calculated as described previously [22].

### siRNA-mediated silencing

Transfection of siRNAs was performed by plating 1x10^5^ cells on 6-well plates and antibiotic free media, one day before transfection. The next day cells were transfected with 25 nM of siRNA and 6 μl of Dharmafect4 according to Dharmacon´s instructions. 24 hours after cells were tripsinized and 5x10^4^ cells plated on transwells in order to perform the transendothelial permeability assay to dextrans, as described previously. In parallel the same number of cells was plated on gelatin-coated 96 well-plates. These were treated in the same way as cells on transwells and lysed on RIPA buffer at the end of the experiment for immunobloting analysis of protein expression.

### Immunobloting

Lysates were run on a SDS–PAGE gel using 8–12% polyacrylamide gels. Proteins were transferred onto nitrocellulose membranes and blocked for 1 h with 5% BSA. Primary antibodies were incubated overnight at 4°C, and secondary antibodies conjugated with horseradish peroxidase were incubated for 1 h at room temperature. Membranes were visualized using Pierce ECL Western Blotting Substrate (Thermo Scientific).

### Microscopy

For the immunolabeling of VE-cadherin and ZO-1 proteins, HUVECs were plated on top of gelatin-coated glass coverslips and treated the same way as cells on transwells. At the end of the assay cells were washed twice with PBS containing Ca^2+^ and Mg^2+^ and fixed with 4% paraformaldehyde (PFA) in PBS for 20 min at room temperature. Immunolabeling was performed overnight at 4°C using 1:100 dilution of the primary antibodies. After washing three times with PBS, labeling with the secondary antibody, Alexa Fluor 488, at 1:500 dilution in PBS was performed for 1 hour at room temperature. Coverslips were then mounted on top of DAPI containing VECTASHIELD. Epi-fluorescence images of immunolabeled cells were acquired using a widefield fluorescence microscope (Leica DM 5000B). The visualization of intracellular FITC dextrans was performed on HUVECs plated on 6.5 mm diameter inserts and 0.4 μm pores from Corning and treated as described for the other experiments. At the end of the assay cells were washed twice with PBS containing Ca^2+^ and Mg^2+^ and fixed with 4% PFA in PBS for 20 min at room temperature Filters were then mounted on a glass slide on top of a drop of DAPI containing VECTASHIELD and covered with a coverslip. Cells were then observed on widefield fluorescence microscope (Leica DM 5000B). For the colocalization experiments cells grown of filters as previously described for the transendothelial permeability assay, were incubated together with 1 mg/ml of 70 kDa FITC-dextrans and 20 μg/ml cholera toxin subunit B (CTxB) for 15 minutes. Cells were then washed three times with PBS containing Ca^2+^ and Mg^2+^ and finally fixed with 4% PFA in PBS overnight at 4°C. Filters were mounted on a glass slide on top of a drop of DAPI containing VECTASHIELD and covered with a coverslip and observed on a Zeiss 710 confocal point-scanning microscope using a 63x Plan-Apochromat objective.

### Image analysis

The number of intercellular gaps (absence of ZO-1 staining at cell-to-cell contacts), of dextran-containing vesicles and colocalization events were scored manually and in a blind manner.

### Statistics

Results are expressed as averages ± standard deviation values relative to controls from at least three independent experiments. Significance values have been calculated using a two-tailed unpaired student´s *t* test at the 95% confidence interval (* *P*<0.05).

## Results

**Fig 1:**
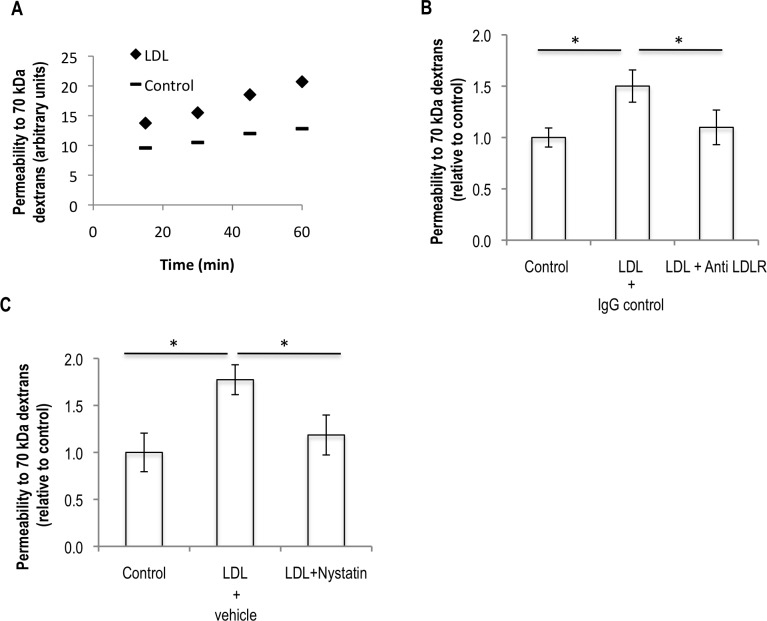
LDL increases endothelial permeability in an LDLR and cholesterol-dependent way. (A) HUVECs were plated on top of 0.4 μm pores size transwell inserts and cultured in order to form a confluent and mature monolayer. Cells were either incubated with 100 μg/ml LDL or with the same volume of the control buffer. 24 hours later, cells were washed with serum-free media and the permeability of the monolayer to 70 kDa FITC-dextrans was assessed two hours later, by measurement of the fluorescence at the bottom chamber of the culture system. (B) The same experiment as in (A) with the addition of 2 μg/ml of anti-LDLR or the IgG control at day 7, one hour before the addition of LDL. Fluorescence at the bottom chamber was measured 15 minutes upon the addition of the dextrans. (C) The same experiment as in (B) with the addition of 50 μg/ml of nystatin or the same volume of vehicle at day 8, one hour before the addition of LDL. All the data, except from panel (A), which is a representation of an experiment performed twice with similar results, represent the averages ± standard deviation of at least three independent experiments. Significance values have been calculated using a two-tailed unpaired student *t* test at the 95% confidence interval (* *P*<0.05).

To assess the effect of LDL on endothelial permeability we performed an *in vitro* permeability assay using 70 kDa dextrans. For this, confluent HUVECs were cultured on top of 0.4 μm pore size transwells in the presence of 100 μg/ml LDL or the control buffer for 24 hours. To control for a possible effect of the transport of LDL itself on the permeability assay, cells were washed from the LDL and control conditions before addition of the dextrans to the monolayer. In this way we were able to observe that pre-treatment of endothelial cells with LDL for 24 hours leads to a significant increase in endothelial permeability to 70 kDa dextrans (**Fig 1A**). To test whether this effect was dependent on the action of the LDLR we tried to revert the phenotype by blocking the binding of LDL to the LDLR. As this, using an antibody against the LDLR added one hour before the addition of LDL, was able to fully revert the permeability phenotype **(Fig 1B)**. To see whether cholesterol was involved in this phenotype, nystatin was used to sequester cholesterol from membranes and it was able to fully revert the increase in permeability suggesting that LDL increases endothelial monolayer permeability in a cholesterol-dependent manner (**Fig 1C**).

**Fig 2:**
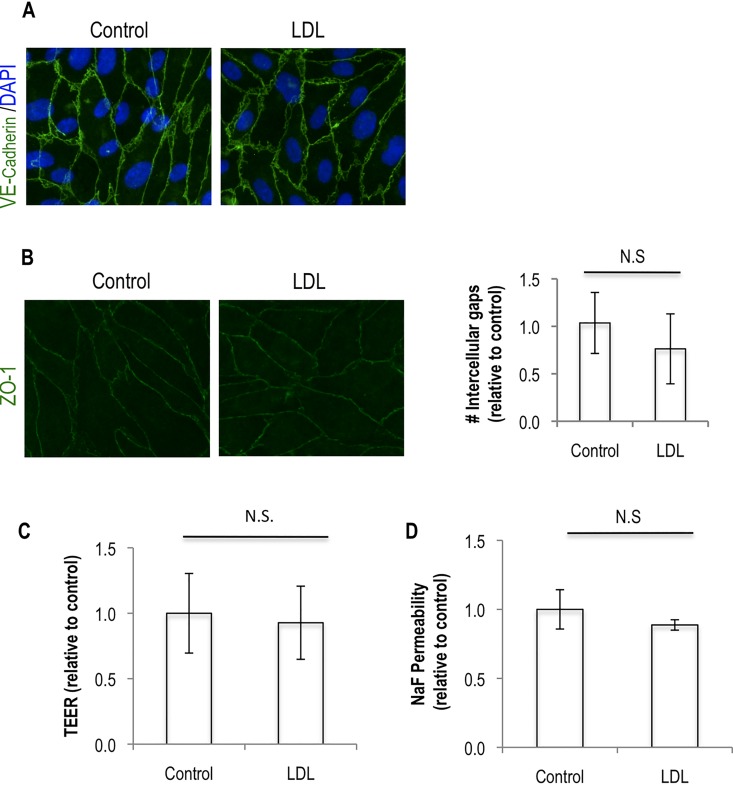
The effect of LDL on endothelial permeability is not caused by disruption of junctions. (A) Immunofluorescence for the junctional proteins VE-cadherin and (B) ZO-1, two hours after washing from control and LDL conditions. The number of intercellular gaps (absence of ZO-1 staining at cell-to-cell contacts) per field was quantified in 10 different fields of view (A—right panel). (C) TEER assay two hours after washing from control and LDL conditions. (D) Permeability assay to sodium fluorescein (NaF) two hours after washing from control and LDL conditions. All the data represent the average ± standard deviation of at least three independent experiments. Significance values have been calculated using a two-tailed unpaired student’s *t* test at the 95% confidence interval (* *P*<0.05).

To understand whether the paracellular or transcellular transport was being affected by LDL, we first looked at the localization of adherens and tight junction proteins at sites of cell-to-cell contact. The localization of the adherens junction protein vascular endothelial (VE)-cadherin and the tight junction protein Zonula Occludens (ZO)-1 at sites of cell-to-cell contacts was however not affected by the addition of LDL to the endothelial monolayer (**Fig 2A and 2B**). This suggested that LDL was increasing permeability without affecting the paracellular route. However as alterations at endothelial permeability at the level of the paracellular route can go together with no major changes at the localization of junctional proteins, we measured the transendothelial electrical resistance (TEER) of the monolayer in the presence of LDL. Treatment of endothelial cell monolayers with LDL did not significantly alter the TEER values of the monolayer (**Fig 2C**). The same was seen for the passage of sodium fluorescein (NaF) (**Fig 2D**). Put together these data show that in our experimental conditions the paracellular route was not being affected by LDL and suggested that the increased transport of 70 kDa molecules was occurring via transcytosis.

**Fig 3:**
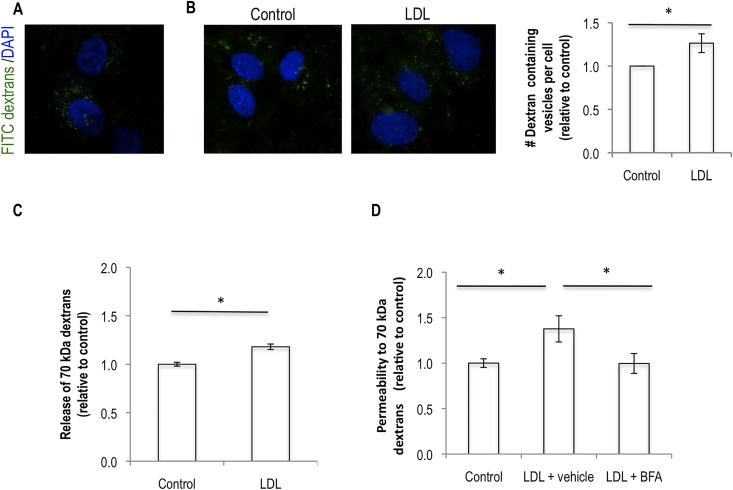
LDL favors the transcytosis of high molecular weight dextrans. The same experiment as in Fig 1A was performed. (A) At the end of the experiment, cells were washed twice with PBS and fixed with PFA. A confocal image showing dextrans inside cells is presented. (B) The number of dextran-containing vesicles per cell, two hours after washing from control and LDL conditions was assessed by widefield fluorescence microscopy. Representative images of each condition are shown and the chart represents the quantification of three independent blind experiments in which 50 cells were analyzed. (C) The same experiment as in Fig 1A with addition of 70 kDA dextrans for 15 minutes, followed by three washes and re-incubation with fresh media both at the top and bottom chambers, 15 minutes later a sample from the bottom chamber was collected and analyzed. (D) The same experiment as in Fig 1A with the addition of either vehicle or 5 μg/ml of Brefeldin A (BFA). Significance values have been calculated using a two-tailed unpaired student *t* test at the 95% confidence interval (* *P*<0.05).

**Fig 4:**
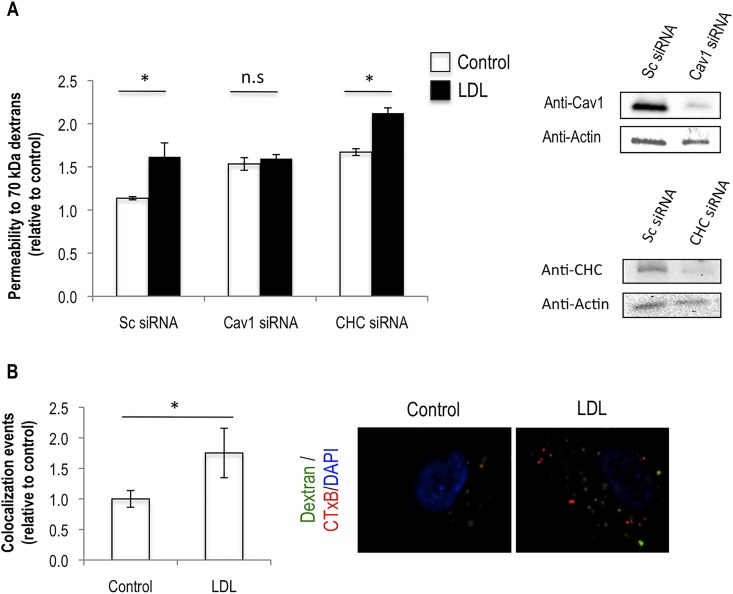
Knock down of Caveolin 1 reverts the effect of LDL on endothelial permeability and 70 kDa dextrans colocalize with cholera toxin subunit B. (A) HUVECs were transfected with either scramble siRNA (Sc siRNA) or siRNAs for Caveolin1 (CAV1) and Clathrin Heavy Chain (CHC) and the day after were either plated on top of 0.4 μm pores size transwell inserts or on 96 well plates. Cells were then cultured as in Fig 1A. At the end of the experiment cells on transwells were used for the permeability assay to 70 kDa FITC-dextrans (left panel). Cells on 96 well plates were lysed and used to check for protein levels by immunobloting (right panel). (B) HUVECs grown on transwells and cultured as in Fig 1A were incubated at the same time with 70 kDa FITC-dextrans (1 mg/ml) and Alexa594-cholera toxin subunit B (CTxB, 20 μg/ml) for 15 minutes. Transwells were then fixed and analyzed by confocal microscopy (63X magnification) for the presence of colocalization events. The graphs represent the average +/- the standard deviation of either the number of colocalization events (top-left panel), or the number of CTxB-containing vesicles (top-right panel) present in control and LDL-treated cells. Representative images of three independent experiments in which 30 randomly selected cells have been analyzed, per condition, are shown (lower panel). Significance values have been calculated using a two-tailed unpaired student *t* test at the 95% confidence interval (* *P*<0.05).

To confirm this hypothesis we checked whether the 70 kDa dextran molecules were internalized by endothelial cells and whether this was enhanced by exposing cells to LDL. Confocal imaging through the middle of endothelial cells showed the presence of FITC-70 kDa dextrans inside endothelial cells and this was increased by LDL treatment (**Fig 3A and 3B**). Additionally we showed that LDL also favours dextran release to the basal side of the monolayer after its removal from the apical side, suggesting dextrans are being internalized and then released (**Fig 3C**). We also show that inhibiting trafficking from the Golgi (using Brefeldin A), known to be involved in transcytosis, also inhibits the effect of LDL on endothelial permeability to the 70 kDa dextrans (**Fig 3D**). Finally, to gain further insights on how LDL would be increasing transcytosis we investigated whether LDL was affecting any of the two main transcytosis pathways that occur in endothelial cells: caveolae and/or clathrin-coated pit mediated. We performed siRNA mediated silencing of Caveolin1 and Clathrin Heavy Chain, respectively. From such experiment we were able to observe that silencing of Caveolin1 rescues the LDL-induced increase in endothelial permeability. The same was not observed when Clathrin Heavy Chain protein levels were knocked down (**Fig 4A**). This result suggests that LDL is increasing the transcytosis of molecules through endothelial monolayers via caveolae-mediated trafficking. In agreement with this we were able to detect colocalization of 70 kDa FITC dextrans with cholera toxin subunit B—CTxB (a marker of caveolae-mediated transcytosis) and that this is increased in the presence of LDL (**Fig 4B**). Together the data presented here allow us to conclude that LDL affects the transcellular transport of molecules through endothelial cells.

## Discussion

Majority of cells have the ability to synthesize cholesterol, however, most cholesterol in cells originates from the internalization of circulating lipoproteins such as LDL. Apart from studies performed using oxLDL, how cells cope with excessive levels of LDL in their environment is not well understood. Here we address how high nLDL levels affect the barrier function of the endothelia. Using an *in vitro* permeability system and the blockage of LDL binding with the LDLR using a specific antibody against the receptor. We were able to show that LDL increases endothelial permeability in a LDL receptor-dependent manner.

Pathological increases of endothelial permeability are known to occur and cause problems such as edema or dissemination of cancer cells to form metastasis [23,24,25]. In many cases, alterations in endothelial barrier function have been shown to occur by either death of ECs or opening up of the junctions [26]. In fact, others have shown that oxLDL affects the integrity of endothelial junctions and therefore endothelial permeability and that short-term treatment with nLDL has no effect on endothelial permeability [19,27]. Here, using a longer exposure time (24 hours) followed by washing, we were able to see that nLDL increases the transcellular transport of high molecular weight molecules. This shows that LDL exposure induces alterations on endothelial cells that change the level of permeability of the monolayer. These changes last even in the absence of LDL in the media. Because LDL is not present in the media at the time of addition of dextrans we rule out the hypothesis that these are being transcytosed together with LDL.

Contrary to what happens for the paracellular route, the way transcytosis may be modulated by external cues is almost undefined. Here we show that external cholesterol carried by LDL induces increased transcytosis on endothelial cells. Caveolae are the main mediators of transcytosis in endothelial cells, but clathrin coated vesicles and other types of vesicular systems have been described to mediate the transport of cargo through vascular beds [1]. Several of these endocytic pathways have been shown to be dependent of the presence of cholesterol in the membranes; however how exogenous cholesterol affects such pathways and how this may contribute to transcytosis is not well described. Here by knocking down the levels of Caveolin1 protein we were able to rescue the LDL-induced increase in permeability, indicating that LDL increases transcytosis in a caveolae-dependent way. Moreover we detect colocalization of dextrans with CTxB and that this increases with LDL. We were not however able to detect any change on Caveolin1 expression, either at the mRNA or proteins levels (not shown). These results go together with a different report suggesting that LDL treatment increases the binding of Caveolin1 to the plasma membrane of endothelial cells but not it´s expression [28].

Together, our findings highlight the importance of LDL-cholesterol in the regulation of intra-endothelial membrane trafficking and endothelial barrier function. This increases our knowledge of how the transport of cargo through endothelial monolayers is achieved and may also contribute to understand how hypercholesterolemia affects disease.
